# Clinical, Laboratory and Radiographic Features Associated With Prolonged Hospitalization in Children With Complicated Appendicitis

**DOI:** 10.3389/fped.2022.828748

**Published:** 2022-04-06

**Authors:** Jyotsna Bhattacharya, Ellen J. Silver, Einat Blumfield, Dominique M. Jan, Betsy C. Herold, David L. Goldman

**Affiliations:** ^1^Pediatric Infectious Disease, Children’s Hospital at Montefiore, Albert Einstein College of Medicine, Bronx, NY, United States; ^2^Academic General Pediatrics, Children’s Hospital at Montefiore, Albert Einstein College of Medicine, Bronx, NY, United States; ^3^Pediatric Radiology, Children’s Hospital at Montefiore, Albert Einstein College of Medicine, Bronx, NY, United States; ^4^Pediatric Surgery, Children’s Hospital at Montefiore, Albert Einstein College of Medicine, Bronx, NY, United States; ^5^Department of Microbiology and Immunology, Albert Einstein College of Medicine, Bronx, NY, United States

**Keywords:** appendicitis, abscess, imaging, microbiology, antibiotics

## Abstract

**Objective:**

In children with appendicitis, rupture of the appendix is associated with a significant increase in morbidity. We sought to characterize the spectrum of illness in children with complicated appendicitis and to define those factors associated with a longer hospital stay.

**Study Design:**

We conducted a retrospective review of 132 children, 18 years of age or younger at a large urban teaching hospital in the Bronx, NY between October 2015 and April 2018 with an intraoperative diagnosis of perforated appendix. Clinical, laboratory and radiologic findings were reviewed, and the primary study outcome was length of stay (LOS) dichotomized at the median, which was 7 days. Statistical analyses were done to characterize morbidity and define variables predictive of longer stay.

**Results:**

Children in the longer LOS group experienced significantly more morbidity, including ICU stay, ileus, and need for multiple drainage procedures. A longer duration of symptoms prior to presentation was associated with a longer stay. Multivariable logistic regression analysis indicated that the presence of abscess and presence of free fluid in the right upper quadrant (RUQ FF) on initial imaging and C-reactive protein (CRP) level >12 at admission, were independently associated with a longer stay.

**Conclusion:**

There is considerable variation in the morbidity of complicated appendicitis. The association between longer stay and the findings of abscess and RUQ FF on initial imaging along with an elevated CRP may provide a useful tool in identifying those children at risk for worse outcomes.

## Introduction

Acute appendicitis is classified as simple or complicated based on radiographic, intraoperative and/or histologic findings. Complicated appendicitis, which is defined as appendiceal rupture with or without abscess or phlegmon formation, occurs in as many as 30% of children who present with acute appendicitis and is associated with significantly higher morbidity than uncomplicated appendicitis (1, 2).

Complicated appendicitis is associated with an increased risk of abscess formation, prolonged hospitalization, and wound infection in the short term (3–5) and small bowel obstruction requiring surgical intervention as a long term complication (6). There has been a growing body of literature around the distinction between complicated and uncomplicated appendicitis to help guide management decisions in children (7, 8).

Despite the higher morbidity of complicated appendicitis in adults, the clinical spectrum of this entity as well as the factors associated with a worse prognosis have not been well characterized in pediatrics. This information could have important implications for optimizing the management of complicated appendicitis in children, which to date remains unclear (9–11). To begin to address this knowledge gap, we conducted a 2.5-year retrospective study to identify factors associated with a prolonged length of stay (LOS), as a surrogate marker for disease severity in children with complicated appendicitis.

## Materials and Methods

### Study Design

We conducted a retrospective review of data from the electronic medical records of children 18 years of age or younger who were hospitalized at the Children’s Hospital of Montefiore (CHAM) between October 2015 and April 2018 with an intraoperative diagnosis of perforated appendix with or without phlegmon or abscess. Patients were excluded if they had surgery or interventional radiology treatment at an outside hospital prior to being transferred to CHAM or were immunocompromised. Patients were also excluded if their operative procedure was delayed more than 48 h from admission. This group consisted of a small number of patients (*n* = 4) whose diagnosis was not initially recognized (see below). The study was approved by the Einstein-Montefiore Institutional Review Board.

### Measures

Demographics, body mass index (BMI), numbers of days of fever and of gastrointestinal (GI) symptoms, admission laboratory findings including white blood cell count (WBC), CRP, sedimentation rate (ESR), treatment modalities (surgery, interventional radiology, and antibiotics), bacterial culture results, and length of stay (LOS) were extracted from the electronic medical record (EMR) over a 2-month period following hospital admission. We also extracted data pertaining to hospital complications including ileus, nasogastric tube (NGT) placement, days to tolerating enteral diet, supplemental oxygen, systemic inflammatory syndrome (SIRS), admission or transfer to the intensive care unit (ICU), and subsequent interventional or surgical procedures. Socioeconomic status (SES), which was calculated from small census tract and block data based on home address, is presented as a z score representing the deviation of this value from the mean of the New York state population (12). Our institution has no standardized protocol for the radiologic evaluation of appendicitis. Ultrasound is the preferred initial method for imaging and if the results are equivocal, a CT or MRI is performed.

### Study Definitions

Body mass index was categorized based on CDC criteria as (i) underweight (<5th percentile), (ii) normal weight (5th–< 85% percentile), (iii) overweight (85–95th percentile), and (iv) obese (≥95 percentile) (13). Hyponatremia was defined as a serum sodium concentration less than 133 mEq/L (14). Systemic inflammatory response syndrome (SIRS) was defined as fever or hypothermia (temperature >38 or <36°C), hypotension for age, requiring fluid resuscitation or treatment with a vasoactive medication (15). Antibiotic mismatch was defined as empiric antibiotic therapy administered for ≥24 h for which bacteria recovered from intraperitoneal (IP) cultures were resistant. Days of GI symptoms referred to days during which patient experienced abdominal pain, vomiting, or diarrhea.

### Radiology Review

A single pediatric radiologist (13 years of experience) independently reviewed all imaging studies obtained during hospitalization including ultrasound (US), computerized tomography (CT), Magnetic resonance imaging (MRI), and radiographs for the following parameters: (i) identification of an appendix; (ii) appendix diameter; (iii) appendicolith; and (iv) presence of free fluid (FF) in the abdomen or pelvis; (v) location of FF, and whether FF was simple or complex; (vi) ileus or small bowel obstruction; (vii) presence of an abscess or phlegmon at the time of initial presentation prior to any intervention; and number of abscesses. Abscess size was recorded as the largest measurement in any one of three dimensions. Phlegmon was defined as the presence of ill-defined edema and inflammation around the appendix without a distinct collection.

### Statistical Methods

Descriptive statistics were used to summarize findings. LOS was dichotomized at the median to categorize patients into two groups for analyses. Chi-square and ANOVA were used for comparisons of categorical variable and continuous variables, respectively, between those with long (=median) and short (< the median) LOS. ROC analyses predicting longer LOS were used to identify optimal cut-offs for continuous independent variables (e.g., symptom days, lab values). Stepwise logistic regression including admission variables having signification associations with LOS in bivariate analyses was performed to identify the factors independently associated with a longer LOS. Data were analyzed using SPSS Version 25 (IBM Corp., Armonk, NY, United States) and tests were two-sided with a significance level < 0.05.

## Results

### Demographics, Clinical Characteristics, and Dichotomization of Cohort Based on Length of Stay

We identified 132 children with complicated appendicitis over the study period of whom 123 (93%) underwent a surgical or interventional procedure within 48 h of admission including open appendectomy (*n* = 73), laparoscopic appendectomy (*n* = 45), or an image guided abscess drainage procedure (*n* = 5). There were no conversions of interventional procedures to open procedures. Four patients underwent procedures > 48 h from presentation (median 120 h) because of delayed diagnosis and five were managed conservatively with antibiotics alone and were scheduled for delayed appendectomy (within 3 months of presentation). These nine were excluded from further analyses because their management was fundamentally different, and the groups were too small for statistical comparisons.

Patient demographics are summarized in Table 1. The mean age of the total study population was 9.3 years (range 1.6 years–18 years); 26 (21.1%) were ≤ 5 years of age. Sixty-seven (54.4%) of the patients were male, 54.3% identified as Hispanic/Latino, 19% as Black/African American, 6.9% as White, and 19.9% did not self-identify. These demographics are reflective of the overall hospital population. The overall SES z-score was −3.74 and did not differ between Black/African Americans (−3.73 ± 2.67) and Hispanic/Latinos (−4.32 ± 2.54) but was lower in those groups compared to Whites (−0.89 ± 2.36) (*p* = 0.003). Approximately, 14.4% of the cohort was obese and 21.2% was overweight. There were no deaths during the study period.

**TABLE 1:** Demographics of cohort by dichotomized length of hospitalization (LOS).

	Total	LOS ≤ 7	LOS > 7 days	*p-*value*
Age (years)**	9.3 ± 4.0	9.1 ± 3.9	9.6 ± 4.2	0.510
Age groups				0.133
≤5 years (%)	26 (21.1)	15 (20.5)	11 (22.0)	
6–11 (%)	59 (48.0)	40 (54.8)	19 (38.0)	
12–18 (%)	38 (30.9)	18 (24.7)	20 (40.0)	
Sex (Male: Female %)	54.5:45.5	53.4:46.6	56:44	0.778
Race (*n* = 116)				0.006^§^
Hispanic/Latino (%)	63 (54.3)	45 (65.2)	18 (38.3)	
Black/African American (%)	22 (19.0)	8 (11.6)	14 (29.8)	
White (%)	8 (6.9)	2 (2.9)	6 (12.8)	
Other (%)	23 (19.8)	14 (20.3)	9 (19.1)	
SES *z*-score (*n* = 123)**	−3.7 ± 2.7	−3.9 ± 2.9	−3.4 ± 2.4	0.378
BMI categorization (*n*, %)				0.058
Obese (≥ 95th percentile)	17 (14.4)	8 (11.4)	9 (18.8)	
Overweight (85th – <95th percentile)	25 (21.9)	14 (20.0)	11 (22.9)	
Healthy (5th – < 85th percentile)	76 (64.4)	47 (67.1)	23 (47.9)	
Underweight (<5th percentile)	6 (5.1)	1 (1.4)	5 (10.4)	

*P value for comparison between length of stay groups. ** Mean ± standard deviation. ^§^Race: Post hoc analyses showed that the LOS > 7 group has a smaller proportion of Hispanic/Latino patients and a larger proportion who are Black/African American compared to the LOS ≤ 7 group.

The median LOS was 7 days (range 2 – 24 days) with 50 (40.6%) patients dichotomized into the LOS > 7 d group and 73 (58.3%) into the LOS ≤ 7 d group. There were no differences in age, sex, SES or BMI between the two LOS groups, but children with the longer LOS course were more likely to be Black/African American (*p* = 0.014) and less likely to be Hispanic/Latino (*p* = 0.004).

### Clinical Presentation

The most common presenting symptoms were abdominal pain 122/123 (99.2%), vomiting 108/123 (87.8%), and diarrhea 72/123 (41.5%). Most children (*n* = 112, 91.1%) had 2 or more GI symptoms. The average duration of fever was 1.6 ± 1.7 days. The patients with greater LOS had significantly longer duration of fever prior to admission (*p* = 0.013), more days of abdominal pain (*p* = 0.014), days of vomiting (*p* = 0.011), and days of diarrhea (*p* = 0.014) (Table 2). Most patients with complicated appendicitis had elevated inflammatory markers including WBC (median, 17.1 ± 5.7 cells/L) and CRP (median, 13.8 ± 9.0 mg/dL). While there were no differences in mean total WBC or ESR, children with a longer LOS had higher immature neutrophil counts, C-reactive protein and were more likely to present with hyponatremia (Table 2).

**TABLE 2:** Clinical, laboratory and radiographic features of cohort by dichotomized LOS.

	Total	LOS ≤ 7 days	LOS > 7 days	*p* -value*
Duration of symptoms prior to admission**				
Days of fever	1.6 ± 1.7	1.3 ± 1.4	2.0 ± 1.9	0.013
Days of abdominal pain	3.54 ± 3.18	2.96 ± 1.93	4.38 ± 4.31	0.014
Days of vomiting	2.30 ± 1.81	1.96 ± 1.65	2.80 ± 1.93	0.011
Days of diarrhea	1.23 ± 1.15	0.82 ± 1.25	1.64 ± 2.35	0.014
Days of any GI symptoms	3.7 ± 3.2	3.0 ± 2.0	4.7 ± 4.3	0.004
Initial Labs**				
White blood cell count (cells/L) (*n* = 122)	17.1 ± 5.7	17.1 ± 5.2	17.0 ± 6.4	0.978
Immature neutrophils (cells/uL) (*n* = 54)	1551 ± 1916	1056 ± 1183	2270 ± 2506	0.021
ESR (mm/hr) (*n* = 39)	44.6 ± 30.2	38.79 ± 23.0	50.25 ± 35.5	0.242
CRP (mg/d) (*n* = 60)	13.8 ± 9.0	11.3 ± 8.0	17.0 ± 9.4	0.014
Sodium ≤ 133 (mEq/L) (*n* = 122)	22 (18.0)	9 (12.3)	13 (26)	0.045
**Radiographic findings (US, CT, and/or MRI) (*n* = 107)**				
Appendix size (cm) (*n* = 86)##	13.3 ± 3.1	13.0 ± 3.1	13.5 ± 3.1	0.457
Appendicolith (*n* = 91, %)	53 (58.2)	27 (54)	26 (63.4)	0.365
Free Fluid (*n* = 101, %)	84 (83.2)	47 (82.5)	37 (84.1)	0.828
RUQ FF (*n* = 100, %)	35 (35.0)	14 (25.5)	21 (46.7)	0.027
RLQ FF (*n* = 101, %)	76 (75.2)	40 (71.4)	36 (80.0)	0.321
Pelvic FF (*n* = 97, %)	48 (49.5)	27 (49.1)	21 (50)	0.929
Complex free fluid (*n* = 84, %)	39 (46.4)	19 (41.3)	20 (52.6)	0.300
Abscess (*n* = 97, %)	33 (34.0)	10 (18.5)	23 (53.5)	<0.001
Abscess size (cm)^#^ (*n* = 33)	5.6 ± 2.4	5.7 ± 2.2	5.5 ± 2.6	0.873
Phlegmon (*n* = 92, %)	76 (82.6)	41 (82.0)	35 (83.3)	0.867
Ileus/Small bowel obstruction (*n* = 44, %)	23 (52.3)	8 (38.1)	15 (65.2)	0.072

*P value for comparison between LOS groups. ** Mean ± standard deviation. ^##^Abscess size in cm represents the longest measurement in any direction.

### Radiologic Findings

Imaging for suspected appendicitis was conducted in 107/123 (87%) of the cohort to confirm the diagnosis prior to surgery. The most common single modality was ultrasound (*n* = 63, 58.9%) followed by computerized axial tomography (CT) (*n* = 12, 11.2%). The others had more than one imaging study performed with 26 (24.3%) patients having both United States and CT, 5 (4.7%) United States and magnetic resonance imaging (MRI), and 1 (0.9%) patient underwent all three imaging modalities. Findings are summarized in Table 2 with the most common findings being free fluid (83.2%), phlegmon (82.6%), and appendicolith (58.2%). The radiographic findings that were predictive of longer LOS were the presence of an intra-abdominal abscess (53.5% vs. 18.5%, *p* <0.001) and the presence of free fluid in the right upper quadrant (RUQ FF) (46.7% vs. 25.5%, *p* = 0.027).

### Microbiology and Antibiotic Treatment

**FIGURE 1:**
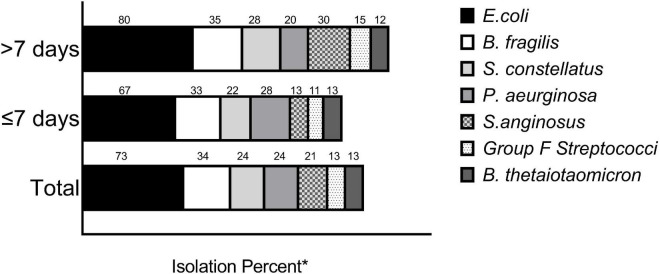
Intraperitonal culture results. Shown (above the bars) are the percentages of the most common bacterial species isolated from intraperitoneal cultures by LOS cohort. *The numbers do not add up to 100 percent as many of the cultures were polymicrobial.

Sixty-four patients (52%) had at least one blood culture obtained during admission and 2 of these 64 (3.1%) had documented bacteremia, one with *Escherichia coli* and one with *Streptococcus constellatus*. Ninety-four patients had peritoneal cultures obtained intraoperatively and 86 (91.5%) of these cultures yielded bacterial growth (Table 3). Most cultures were polymicrobial with the four most common isolates being *E. coli*, *Bacteroides fragilis*, *S. constellatus*, *Pseudomonas aeruginosus*, and *S. anginosus* (see Figure 1). Empiric antibiotics administered on admission included piperacillin-tazobactam (56.1%), cefoxitin (18.6%), ceftriaxone and metronidazole (13%), and ceftriaxone alone (4.0%). Antimicrobial mismatch to the empiric regimen based on culture results was detected in 15 (17.4%) of patients and were associated with isolation of the following bacteria: multi-drug resistant *E. coli* (*n* = 3), extensively drug resistant *E. coli* (*n* = 1), vancomycin resistant enterococci (*n* = 2), AmpC beta-lactamase producing Enterobacteriaceae (*n* = 3), methicillin-resistant *Staphylococcus aureus* (*n* = 1), and *P. aeruginosa* (*n* = 5).

**TABLE 3:** Microbiologic and antibiotic treatment of patients by dichotomized LOS.

	Total	LOS ≤ 7 days	LOS > 7 days	*p-* value*
Cultures obtained in the OR/IR^§^ (%)	94 (76.4)	53 (72.6)	41 (82.0)	0.228
Positive peritoneal cultures^†^ (%)	86 (91.5)	46 (86.8)	40 (97.6)	0.064
Polymicrobial (%)	71 (82.6)	36 (78.3)	35 (87.5)	0.201
**Antibiotic utilization**				
Antibiotic change during admission (%)^¶^	18 (14.6)	4 (8.7)	14 (35.0)	0.003
Empiric antibiotic mismatch (> 24 h) (%)^$^	15 (17.4)	5 (10.9)	10 (25.0)	0.085
Days of antibiotics (IV + PO)**	7.7 ± 3.6	5.7 ± 1.1	10.7 ± 4.0	<0.001
Days of prescribed after discharge**	4.1 ± 5.2	3.0 ± 3.5	5.8 ± 6.6	0.002

^$^Number of patients with a mismatch, defined as empiric antibiotic therapy ≥ 24 h that did not match susceptibilities of recovered intraperitoneal (IP) bacteria. ^¶^Number of antibiotic changes after initial regimen. **Mean ± standard deviation.

### ROC and Multivariable Logistic Regression Analysis Predicting Longer Length of Stay

ROC analyses revealed that pre-admission fever duration for 1 day or more and having any GI symptom for more than 3 days maximized both sensitivity and specificity in predicting longer LOS. Therefore, these cut-offs were applied in multivariable logistic regression analysis predicting longer LOS. Variables included in the regression model were presence of fever prior to admission, GI symptoms = 3 days, hyponatremia, CRP = 12 (16), and presence of abscess and of RUQ FF noted on initial imaging. Regression analysis indicated that the presence of abscess (OR = 3.60; 95%CI = 1.36–9.48; *p* = 0.01) and presence of RUQ FF on initial imaging (OR = 2.68, 95%CI = 1.05–6.79; *p* = 0.038) and CRP > 12 (OR = 2.84; 95%CI = 1.10–7.32; *p* = 0.031) were independently associated with a longer LOS. No other variables were significant.

### Hospital Outcomes

Patients with a longer LOS experienced significantly more morbidity during hospitalization compared to those with a shorter LOS (Table 4), including more than twice the number of days of fever, ileus, need for NGT and more days before tolerating oral diet (all *p* < 0.001). In addition, those with longer LOS were more likely to require oxygen (*p* = 0.03) and to have symptoms consistent with systemic inflammatory response syndrome (*p* = 0.027). Patients with a longer LOS had more than an eightfold increase in admission rates to the PICU when compared to patients with a shorter LOS. Furthermore, a second drainage procedures was necessary in 40% of patients with a longer LOS compared to 0% of patients with shorter LOS (*p* < 0.001). A longer duration of antibiotics (*p* < 0.001) and more than two changes in antibiotic regimens (*p* = 0.003) were also more common in children with a longer LOS.

**TABLE 4:** Clinical course by dichotomized LOS.

	Total	LOS ≤ 7 days	LOS > 7 days	*p-*value*
Days of fever during admission**	2.7 ± 2.6	1.6 ± 1.3	4.2 ± 3.3	<0.001
Clinical ileus or small bowel obstruction	52 (42.3)	19 (26.0)	33 (66.0)	<0.001
Days of ileus/small bowel obstruction**	4.9 ± 3.1	2.2 ± 0.9	6.4 ± 2.8	<0.001
Nasogastric tube placement (%)	31 (25.2)	6 (8.2)	25 (50)	<0.001
Days of nasogastric tube**	4.6 ± 2.7	1.8 ± 0.4	5.3 ± 2.6	0.003
Days until oral diet**	5.2 ± 3.0	3.5 ± 1.2	7.8 ± 2.9	<0.001
Oxygen requirement	17 (13.8)	6 (8.2)	11 (22.0)	0.030
Systemic inflammatory syndrome (SIRS)	25 (20.3)	10 (13.7)	15 (30.0)	0.027
Days of SIRS**	1.6 ± 1.0	1.1 ± 0.3	2.0 ± 1.2	0.003
Admission/transfer to intensive care (%)	7 (5.7)	1 (1.4)	6 (12.0)	0.012
Days in intensive care unit	2.43 ± 0.79	2	2.5 ± 0.84	0.604
Repeat surgical or interventional radiology	20 (16.2)	0 (0)	20 (40)	<0.001

*P value for comparison be LOS cohorts. **Mean ± standard deviation.

## Discussion

Rupture of the appendix is a serious and potentially life-threatening complication of appendicitis. Our findings indicate that children with complicated appendicitis exhibit significant variation in their disease course, including a wide range in their needs for admission to the PICU and for a second drainage procedure. Dichotomization of the cohort of children admitted to the hospital with complicated appendicitis by median LOS allowed us to characterize the spectrum of illness associated Length of stay in complicated appendicitis may in-fact be seen as an indicator for how well a hospital system identifies and manages available data of at-risk individuals. with complicated appendicitis and to identify those features associated with a worse outcome.

The only demographic feature associated with LOS was race, with Black children were more likely to have a longer LOS compared to Hispanic/Latino children. These findings are consistent with a retrospective study by Pathak et al., who also found a lower risk for prolonged LOS in Hispanic children with perforated appendix (17). The reasons for these differences are not clear. Possibly the differences reflect a delay in seeking or access to healthcare, which has been suggested in other studies (2, 18, 19). Consistent with this, we observed a trend toward a longer duration of GI symptoms prior to admission in Black compared to Hispanic/Latino children (data not shown), which was one of the clinical features associated with LOS. Obesity has previously been associated with delay in diagnosis and increased risk of post-operative complications, including wound infection and dehiscence (20–22). In our study children with BMIs greater than the 85*^th^* percentile had higher oxygen requirements and more episodes of sepsis; however, this did not result in overall longer LOS.

Using bivariate analysis, the admission clinical and laboratory features associated with greater LOS were symptom duration (fever and GI symptoms), immature neutrophils, elevated CRP, and hyponatremia. These laboratory markers are consistent with prior studies that were designed to distinguish between simple and complicated appendicitis (14, 23, 24). The longer duration of symptoms is also consistent with studies showing higher complication rates among children with delayed presentation and treatment for appendicitis (25–27). However, the differences in symptom duration between the groups was small and neither these measures nor admission laboratory values other than CRP > 12 mg/dL predicted longer LOS in the logistic regression analysis.

The only additional predictive factors in the multivariable analysis were detection of an intra-abdominal abscess and the presence of right upper quadrant free fluid in initial imaging. In earlier studies, one of our authors (EB) demonstrated the utility of sonographic findings as part of a clinical score to distinguish between perforated and non-perforated appendicitis (7). Our current findings further highlight the utility of radiologic studies in predicting disease outcome in children with complicated appendicitis. We hypothesize that the presences of organized inflammation in the form of abscess, reflects a later stage of disease in appendicitis and is consistent with worse outcomes in these patients. This effect appeared to be regardless of abscess size,

The peritoneal culture results of complicated appendicitis have been noted to differ compared to uncomplicated appendicitis with increased prevalence of gram positive and anaerobic bacteria (28). There was no significant difference in the species of bacteria isolated for intraoperative cultures based on length of stay. However, we did observe a trend toward a higher rate of isolation of *S. anginosus* species in patients with longer LOS (30 vs. 13% *p* = 0.054, Figure 1). The *S. anginosus* group of bacteria, which includes *S. anginosus*, *S. constellatus*, and *S. intermedius*, are part of the normal gut microbiome but have been associated with abscesses in a variety of organ systems (29). The association of *S. angiosus* group with abscess is incompletely understood but may be attributed to the production of hydrolytic enzymes and hydrogen peroxide by these bacteria which can interfere with neutrophil function and possibly promote neutrophil death (30, 31). Isolation of *S. anginosus* group has been previously associated with the risk of developing a post-operative phlegmon or abscesses and prolonged hospital stay in other studies (32, 33). An unanticipated observation in the microbiological studies was the isolation of *Pseudomonas aeruginosa* in peritoneal cultures in 24% of the patients, although this was not associated with increased LOS. The isolates were susceptible to piperacillin-tazobactam, which was commonly prescribed on admission.

Antibiotic mismatches occurred in approximately 17% of the entire cohort due to the presence of resistant bacteria. Although not significant, a trend toward a higher rate of antibiotic mismatch was noted in the longer LOS group (*p* = 0.085). The role of intraperitoneal cultures in the management of complicated appendicitis has not been fully defined, though several studies highlight its utility (34–39). The most recent IDSA guidelines for the diagnosis and management of complicated intra-abdominal infections, which was published in 2010, distinguishes the need for routine peritoneal culture acquisition based on the risk of antibiotic resistance (34). For high-risk patients, cultures from the site of infection are recommended, chiefly in patients with prior antibiotic exposure, who are more likely than other patients to harbor resistant pathogens. Nonetheless, these guidelines also stipulate that in the context of significant resistance (i.e., resistance in 10–20% of isolates) of a common community isolate (e.g., *Escherichia coli)* to an antimicrobial regimen in widespread local use, routine culture and susceptibility studies should be obtained for perforated appendicitis and other community-acquired intra-abdominal infections. A significant rise in the level of antimicrobial resistant among community acquire infection has occurred since the publication of these guidelines including at our hospital where only 53% of outpatient urinary isolates are susceptible to ampicillin/sulbactam (personal data). Our data regarding antibiotic mismatch and LOS support the potential utility of intraoperative cultures in the management of complicated appendicitis in areas with high levels antimicrobial resistance within the community.

Our study has several limitations including its retrospective nature and the fact that almost all of the patients received prompt surgical intervention, thus limiting the ability to address the question of delayed surgical intervention. Not all of the patients had the same laboratory or radiographic evaluations, and this may result in an unintended bias. Another potential limitation of our study is the relatively high rate of open procedures at our institution, which may not be reflective of the approach at other institutions. This in turn may affect hospitalization stay.

Despite these limitations, dichotomization by median LOS did identify elevated CRP as well as the imaging findings of RUQ FF and abscess on presentation as predictive of a worse outcome. Our findings also highlight the potential utility of intraoperative cultures. Additional study is clearly needed to confirm these results. We further suggest that the identification of surrogate markers for poor outcome in children with complicated appendicitis will ultimate prove useful in future clinical trials designed to tailor and optimize treatments for children with complicated appendicitis.

## Data Availability Statement

The original contributions presented in the study are included in the article/supplementary material, further inquiries can be directed to the corresponding author.

## Ethics Statement

The studies involving human participants were reviewed and approved by Institutional Review Board of the Albert Einstein College of Medicine. Written informed consent from the participants’ legal guardian/next of kin was not required to participate in this study in accordance with the National Legislation and the Institutional Requirements.

## Author Contributions

JB helped to conceive, design, and carryout the study as well analyze and present the data. ES helped with experimental design and instrumental in data analysis and data presentation. EB helped to carryout the study and data analysis as well as data presentation. DJ helped with data analysis and data presentation. BH and DG helped to conceive and design the study as well as analyzed and present the data, and helped with overall guidance of the study. All authors contributed to the article and approved the submitted version.

## Conflict of Interest

The authors declare that the research was conducted in the absence of any commercial or financial relationships that could be construed as a potential conflict of interest.

## Publisher’s Note

All claims expressed in this article are solely those of the authors and do not necessarily represent those of their affiliated organizations, or those of the publisher, the editors and the reviewers. Any product that may be evaluated in this article, or claim that may be made by its manufacturer, is not guaranteed or endorsed by the publisher.

## Abbreviations

LOS: length of stay
FF: presence of free fluid
RUQ FF: right upper quadrant
BMI: body-mass index
WBC: white blood cell count
CRP: C-reactive protein
CT: computerized tomography
MRI: magnetic resonance imaging
NGT: nasogastric tube
SIRS: systemic inflammatory syndrome
ICU: intensive care unit
SES: socioeconomic status
CHAM: Children’s Hospital of Montefiore.
